# Nutritional Status in a Sample of Patients With β-Thalassemia Major

**DOI:** 10.7759/cureus.27985

**Published:** 2022-08-14

**Authors:** Irene Lidoriki, George Stavrou, Dimitrios Schizas, Maximos Frountzas, Lampros Fotis, Alkistis Kapelouzou, Smaro Kokkota, Barbara Fyntanidou, Katerina Kotzampassi

**Affiliations:** 1 First Department of Surgery, Laikon Hospital, Medical School, National and Kapodistrian University of Athens, Athens, GRC; 2 Department of Surgery, AHEPA University Hospital, Aristotle University of Thessaloniki, Thessaloniki, GRC; 3 First Propaedeutic Department of Surgery, "Hippocration" General Hospital, National and Kapodistrian University of Athens School of Medicine, Athens, GRC; 4 Third Department of Paediatrics, "Attikon" University Hospital, National and Kapodistrian University of Athens, Athens, GRC; 5 Clinical, Experimental Surgery and Translational Research, Biomedical Research Foundation Academy of Athens, Athens, GRC

**Keywords:** vitamin d, body composition, nutritional assessment, s:malnutrition, screening of nutritional status, β-thalassemia

## Abstract

Background: Patients suffering from thalassemia have decreased levels of lean body mass and an increased nutritional risk. To assess the body composition and vitamin D levels of thalassemic patients in relation to nutritional risk.

Methods: A total of 67 consecutive adult patients who were diagnosed with thalassemia major and followed a regular blood transfusion scheme were included in this study. Demographic and clinical data were collected for each participant. Blood samples were collected to assess 25-hydroxy-vitamin D (25-OH-D) levels. The assessment of patients’ nutritional risk was based on the Malnutrition Universal Screening Tool. Body composition assessment was based on bioelectrical impedance analysis (BIA).

Results: Eleven patients (16.4%) and five patients (7.5%) were at moderate and high risk for malnutrition, respectively. Moreover, 86.6% of patients had a low fat-free mass index (FFMI) and 74.6% of patients had a high-fat mass (FM) index. The prevalence of sarcopenic obesity and 25-OH-D deficiency was 64.2% and 92.2%, respectively. Medium and high-risk patients had significantly lower BMI (18.81 ± 1.29 vs 23.90 ± 2.65 kg/m^2^, p<0.001), lower FFM index (12.80 ± 1.38 vs 14.19 ± 1.89 kg/m^2^, p=0.009) and lower FM index (5.97 ± 1.86 vs 9.70 ± 2.70 kg/m^2^, p<0.001) than their low-risk counterparts.

Conclusions: Adult patients with β-thalassemia major had low levels of vitamin D and altered body composition, presenting with increased adiposity, low levels of lean body mass, and high rates of sarcopenic obesity. Timely detection of patients at risk could lead to the prioritization of patients who could benefit from nutritional interventions.

## Introduction

Thalassemias are severe inherited anemias characterized by microcytic, hypochromic, and short-lived red blood cells due to defective hemoglobin synthesis. Thalassemias affect mainly people in the Mediterranean region, but they also occur in populations living in the Middle East, Transcaucasia, Central Asia, the Indian subcontinent, the Far East, and Africa [1]. β‐thalassemias are a heterogeneous group of hereditary hemoglobinopathies characterized by defects in the β‐globin chain of hemoglobin and belong to autosomal recessive disorders [2]. Worldwide, approximately 1.5% of people are β‐thalassemia carriers [3], but great variations are observed even within small geographic regions. β-thalassemia major is a severe transfusion-dependent anemia associated with lower survival rates compared to the general population [4]. Although survival and complication‐free survival rates have substantially improved during the last decades due to better treatment options, heart disease (52.3%) and liver carcinoma (13.8%) are still accounting for the majority of β‐thalassemia-related deaths [5]. Multiple transfusions lead to secondary hemochromatosis due to iron overload affecting multiple organs, such as the heart, liver, and endocrine glands, which seem to be the culprit behind the increased morbidity of these patients [6].

Adult patients suffering from thalassemia major have impaired body composition, especially in terms of whole-body lean mass and bone mineral density, as suggested by recent studies [7,8]. Improvements in chelation therapy have been consistently reported to decrease the rate of new endocrine disorders, whereas patients who present poor compliance with chelation therapy are at an increased risk of developing endocrine disorders that affect body composition status [9]. Moreover, impaired nutritional status has been commonly observed in adult patients with thalassemia major, especially in terms of hidden malnutrition (i.e., micronutrient deficiencies), although underweight is not necessarily present. Specific nutritional deficiencies, such as vitamin D, zinc, and iron deficiency, are also often observed due to increased losses of micronutrients or increased endogenous requirements [10].

Nevertheless, little is known regarding the nutritional status of adult patients with thalassemia major and its association with body composition parameters. The aim of the current study was to assess patients’ body composition and vitamin D levels as well as to investigate potential associations between these parameters and patients’ nutritional status.

This article was previously posted to the ResearchSquare preprint server on July 06, 2022.

## Materials and methods

Study sample

Sixty-seven (67) consecutive adult patients (≥18 years old) who were diagnosed with thalassemia major and followed a regular blood transfusion scheme every 10 days at AHEPA University Hospital were eligible for enrollment in this observational study. All patients received subcutaneous iron chelation therapy. Patients with diabetes mellitus, severe cardiomyopathy, severe liver disease, or patients with prolonged immobility were excluded from the study. Participants were prospectively recruited from January 2018 to December 2018. Written informed consent was obtained from all participants prior to enrollment in the study. The study protocol was approved by the Human Research Ethics Committee of AHEPA University Hospital of Thessaloniki, Greece (271/Apr 6, 2012).

Data collection

Demographic characteristics and clinical data were collected for each participant. Body weight and height were also recorded, with all patients being barefoot and in their underwear. Body height was measured to the nearest 0.5 cm using a stadiometer (SECA 222; SECA GMBH & Co, Hamburg, Germany) with their weight evenly distributed on both feet after a deep inhalation. Body weight was measured on an electronic digital scale to the nearest 0.1 kg (SECA 813; SECA GMBH & Co) and BMI was calculated for each patient according to the following formula: BMI=weight (kg)/height (m)^2^. Patients were classified by BMI according to World Health Organization criteria [11]. Blood samples were collected in order to assess 25-hydroxy-vitamin D (25-OH-D levels) [12].

The assessment of patients’ nutritional risk was based on the Malnutrition Universal Screening Tool (MUST). MUST is a simple five-step screening tool used to identify patients who are malnourished and at risk of malnutrition. Patients’ classification relies on BMI, unintentional weight loss in the past three to six months, and acute disease effects or being unable to receive food for more than five days. Patients were classified into one of the basic nutritional status categories as follows: 0 = low risk (well-nourished), 1 = moderate risk, and 2 or more = high risk for malnutrition (malnourished). According to MUST, patients were considered to be at high risk if they had a BMI <18.5, had experienced >10% unintentional weight loss in the previous three to six months, or had no nutritional intake for >5 days. They were considered to be at moderate risk if they had a BMI of 18.5-20.0 or had experienced a 5-10% weight loss in the previous three to six months. All other patients were classified as low-risk [13].

Body composition assessment was based on bioelectrical impedance analysis (BIA), which was conducted once on each patient in the morning before transfusion therapy. A single tetrapolar measurement was applied on the right side of the body using four surface electrodes (Bodyscout; Fresenius Kabi AG, Hamburg, Germany), two of which were attached to the dorsal region of the hand and two to the dorsal region of the foot. Then, a brief, low-density electrical current (50 kHz) was introduced into the body after which tissue resistance was measured, enabling the calculation of low (lean mass) and high-resistance (fat) tissues. Multiple frequencies enabled the estimation of both intracellular fluid and extracellular fluid separately. Fat-free mass (FFM) was calculated by using a previously validated multiple regression BIA equation [14]. Moreover, the results for the resistance (ohm), reactance (ohm), and phase angle (degrees) were directly displayed on the BIA device and transferred to a database. In order to adjust for differences in body height, the absolute values (kg) of FFM and fat mass (FM) were transformed into the fat-free mass index (FFMI) and fat mass index (FMI) by dividing FFM or FM (kg) respectively by their squared body height (m^2^). The FFMI was considered ‘low’ if <17.4 (men) and <15.0 (women) and ‘normal’ if 17.5-19.7 (men) and 15.1-16.6 (women). Regarding FMI, the FMI was considered ‘normal’ if 2.5-5.1 (men) and 4.9-8.2 (women) and ‘high’ if >5.2 (men) and >8.3 (women) [15,16]. In addition, we defined sarcopenic obesity as the combination of low FFMI and high FMI [17].

Statistical analysis

Descriptive statistics were used to analyze the frequencies, means, and standard deviations (SDs) of the study variables. The Shapiro-Wilk test was used to determine the normal distribution. Subgroup comparisons of categorical variables were assessed by a chi-squared or Fisher’s exact test. The Student’s t-test was used for the comparison of means of continuous variables and normally distributed data. Missing data were excluded from the analysis. For all statistical procedures, a P-value of less than 0.05 was considered significant. All calculations were done using the Statistical Package for Social Sciences (SPSS) (version 20.0; SPSS Inc, Chicago, IL, USA).

## Results

Baseline data of study participants

A total of 67 consecutive patients with transfusion-dependent β-thalassemia major were included in our study. Baseline patient characteristics are summarized in Table 1. The mean age of study participants was 37.22 ± 10.27 years. Fifty-one patients (76.1%) were considered well-nourished, while 11 patients (16.4%) and 5 patients (7.5%) were at moderate and high risk for malnutrition, respectively. Fifty-nine patients (92.2%) were 25-OH-D deficient.

**Table 1:** Baseline characteristics of study participants MUST: Malnutrition Universal Screening Tool; BMI: body mass index; 25-OH-D: 25-hydroxy vitamin D

Characteristic	Patients (n=67)
Age (years)	37.22 ± 10.27
Sex	n (%)
Male	26 (38.8)
Female	41 (61.2)
Splenomegaly	n (%)
Yes	26 (38.8)
No	41 (61.2)
Splenectomy	n (%)
Yes	19 (28.4)
No	48 (71.6)
MUST	n (%)
Low risk	51 (76.1)
Medium risk	11 (16.4)
High risk	5 (7.5)
BMI (kg/m^2^)	22.68 ± 3.23
BMI category	n (%)
Underweight	5 (7.5)
Normal weight	47 (70.1)
Overweight	14 (20.9)
Obese	1 (1.5)
25-OH-D (ng/mL)	17.65 ± 9.21
25-OH-D (n=64)	n (%)
Low (<30 ng/mL)	59 (92.2)
Normal (30-150 ng/mL)	5 (7.8)

Body composition of study participants

Patients’ body composition characteristics are presented in Table 2. According to body composition analysis, mean body cell mass (BCM) was 24.15% ± 7.89% and mean fat-free mass (FFM) was 61.99% ± 9.35%, whereas mean fat mass was 38.14% ± 9.06%. Moreover, 86.6% of patients had a low FFM index and 74.6% of patients had a high fat mass index. The prevalence of sarcopenic obesity was 64.2%.

**Table 2:** Body composition parameters of study participants BCM, body cell mass; FFM: fat-free mass; TBW: total body water; ICW: intracellular water; ECW: extracellular water; LTM: lean tissue mass; FM: fat mass *Phase angle = arc tangent of (Xc/R)*180°/π

Characteristic	Patients (n=67)
Arm circumference (cm)	26.87 ± 2.98
BCM %	24.15 ± 7.89
FFM (kg)	38.02 ± 7.23
FFM %	61.99 ± 9.35
Fat mass (kg)	24.07 ± 8.55
Fat mass %	38.14 ± 9.06
TBW %	45.24 ± 6.18
ICW %	24.34 ± 4.09
ECW %	20.91 ± 2.45
LTM %	47.16 ± 11.22
Resistance (Ω)	699.31 ± 103.01
Reactance (Ω)	60.32 ± 11.25
Phase angle (°)*	5.06 ± 0.77
FFM index (kg/m^2^)	13.86 ± 1.87
FFM index (kg/m^2^)	n (%)
Low	58 (86.6)
Normal	9 (13.4)
FM index (kg/m^2^)	8.81 ± 2.98
FM index (kg/m^2^)	n (%)
High	50 (74.6)
Normal	17 (25.4)
Sarcopenic obesity	n (%)
Yes	43 (64.2)
No	24 (35.8)

Association between nutritional status and patients’ characteristics

Patients’ body composition characteristics and vitamin D levels according to their nutritional status are presented in Table 3. Medium and high-risk patients had significantly lower BMI values (18.81 ± 1.29 vs 23.90 ± 2.65 kg/m^2^, p<0.001), lower fat mass percentage (31.52 ±8.82 vs 40.22 ± 8.16 %, p<0.001), lower FFM index (12.80 ± 1.38 vs 14.19 ± 1.89 kg/m^2^, p=0.009) and lower FM index (5.97 ± 1.86 vs 9.70 ± 2.70 kg/m^2^, p<0.001) than their low-risk counterparts. Among low-risk patients, 38 patients (74.5%) were sarcopenic obese, whereas the prevalence of sarcopenic obesity was 31.2% in medium/high-risk patients (p=0.003).

**Table 3:** Participants’ characteristics according to MUST categories ΒΜΙ: body mass index; BCM: body cell mass; FFM: fat-free mass; TBW: total body water; ICW: intracellular water; ECW: extracellular water; LTM: lean tissue mass; FM: fat mass; 25-OH-D, 25-hydroxy vitamin D *Phase angle = arc tangent of (Xc/R)*180°/π

Characteristic	Low risk patients	Medium/high-risk patients	p-value
Age (years)	37.8 ± 9.3	35.3 ± 13.9	0.398
Sex	n (%)	n (%)	0.428
Male	19 (37.3)	7 (43.8)	
Female	32 (62.7)	9 (56.2)	
Splenomegaly	n (%)	n (%)	0.343
Yes	21 (41.2)	5 (31.2)	
No	30 (58.8)	11 (68.8)	
Splenectomy	n (%)	n (%)	0.440
Yes	16 (31.4)	4 (25.0)	
No	35 (68.6)	12 (75.0)	
BMI (kg/m^2^)	23.90 ± 2.65	18.81 ± 1.29	<0.001
Arm circumference (cm)	27.84 ± 2.52	23.75 ± 2.08	<0.001
BCM %	22.71 ±7.38	28.76 ± 7.91	0.006
FFM %	59.80 ± 8.16	68.99 ± 9.70	<0.001
Fat mass %	40.22 ± 8.16	31.52 ± 8.82	<0.001
TBW %	43.86 ± 5.60	49.64 ± 6.03	0.001
ICW %	23.38 ± 3.65	27.43 ± 3.96	<0.001
ECW %	20.50 ± 2.32	22.22 ± 2.47	0.013
LTM %	44.11 ± 9.30	56.68 ± 11.62	<0.001
Resistance (Ω)	676.77 ± 93.70	771.17 ± 100.94	0.001
Reactance (Ω)	58.84 ± 11.11	65.06 ± 10.67	0.053
Phase angle (°)*	5.13 ± 0.82	4.83 ± 0.54	0.175
FFM index (kg/m^2^)	14.19 ± 1.89	12.80 ± 1.38	0.009
FFM index (kg/m^2^)	n (%)	n (%)	0.309
Low	43 (84.3)	15 (93.8)	
Normal	8 (15.7)	1 (6.2)	
FM index (kg/m^2^)	9.70 ± 2.70	5.97 ± 1.86	<0.001
FM index (kg/m^2^)	n (%)	n (%)	<0.001
High	45 (88.2)	5 (31.2)	
Normal	6 (11.8)	11 (68.8)	
Sarcopenic obesity	n (%)	n (%)	0.003
Yes	38 (74.5)	5 (31.2)	
No	13 (25.5)	11 (68.8)	
25-OH-D (ng/mL)	16.86 ± 8.29	20.14 ± 11.98	0.256

## Discussion

The present study investigated the nutritional status and body composition of patients suffering from transfusion-dependent β-thalassemia major. According to our results, the majority of patients (76.1%) were well-nourished, while 16.4% and 7.5% of patients were at moderate and high risk for malnutrition, respectively. Although data regarding the nutritional status of adult thalassemic patients is rather scarce, it is speculated that patients with thalassemia may be at increased risk for nutritional deficiencies due to elevated nutrient requirements and/or insufficient intake and absorption of nutrients. More specifically, Fung et al. demonstrated that the typical dietary intake of patients with thalassemia was more frequently inadequate compared to control subjects [18]. Another study revealed that more than 30% of thalassemic subjects consumed inadequate levels of vitamin A, D, E, K, folate, calcium, and magnesium [10]. The exact reason behind inadequate intake of nutrients has yet to be determined, but it is supposed that comorbidities and treatments’ side effects contribute to insufficient nutrient intake [10].

Nonetheless, many individuals who reported adequate dietary intake had deficient levels of circulating nutrients, which in turn implies causes of nutrient excretion or loss, in addition to higher micronutrient requirements [10]. For example, due to iron’s molecular similarity to zinc and copper, it is likely that these nutrients may be chelated instead of, or along with, iron. Moreover, vitamin C and zinc levels also have a negative relationship with liver iron concentration. Iron overload increases hepatic metallothionein concentrations, which bind zinc, leading to sequestration. In patients with thalassemia with hyperactive erythropoiesis, folate requirements are also increased and deficiencies are commonly reported [19,20].

On the other hand, 70.1% of our study sample fell into the normal weight category, according to BMI values. The prevalence of underweight was low (7.5%), whereas 22.4% of study patients were overweight or obese. Our results are in accordance with a previous study that assessed body composition in patients with thalassemia. The prevalence of underweight was 5.2% and the prevalence of overweight/obese was 26.5%. The latter was considered low when compared with median data from NHANES 1999-2000, suggesting that thalassemic patients are leaner than their healthy counterparts [18]. However, in another study in patients with thalassemia major, BMI was lower only in male patients compared to healthy age-matched controls, whereas the female patient group exhibited non-statistically significant differences [7]. This inconsistency could be attributed to the fact that BMI is only a crude measure of nutritional and body composition status since it cannot distinguish patients with the same weight but different proportions of lean and fat body mass [21].

For this reason, a more precise assessment of body composition is required, in order to detect patients with high levels of adiposity and/or low fat-free mass. In our study, we used the BIA method to assess the body composition of thalassemic patients. Our analyses demonstrated that almost 87% of patients had a low FFM index, whereas almost 75% of patients had a high-fat mass index. Moreover, a great number of patients (64.2%) were sarcopenic obese, i.e., they had a high-fat mass index in conjunction with a low FFM index. Patients with thalassemia often present with low muscle mass, which might be caused by the low physical activity levels commonly seen in these patients. It is assumed that thalassemic patients have decreased physical activity levels due to severe anemia and cardiomyopathies secondary to iron overload [22]. Sohn et al. assessed the cardiopulmonary function of 71 well-transfused thalassemic patients via a graded treadmill exercise stress test. Their results showed that patients had limited exercise capacity, while patients with low hemoglobin levels had lower O2 pulse and Vo2max, regardless of gender. Moreover, iron toxicity caused by frequent blood transfusions may be related to vascular inflammation and direct modulation of heart rate in response to exercise [23]. Given that physical activity levels are strongly related to body composition, especially lean body mass, it is expected that lack of exercise could lead to reduced muscle mass and increased adipose tissue mass in these patients [18].

Patients with thalassemia tend to have increased adiposity, as has also been reported in previous studies [18,24]. These alterations in body composition are more frequently seen in thalassemic patients compared to the general population and are related to the high prevalence of sarcopenic obesity observed in our study. Sarcopenic obesity is considered to be a major risk factor for increased morbidity and impaired survival in many chronic diseases [25]. Nevertheless, the impact of sarcopenic obesity on thalassemic patients has yet to be determined. Since a considerable number of patients with thalassemia major are diagnosed with metabolic syndrome and other endocrine disorders [26,27], which could be aggravated by sarcopenic obesity, clinicians should focus on the thorough assessment of this group of patients who could benefit from proper dietary consultation and lifestyle changes.

As expected, medium and high-risk patients, according to MUST, had significantly lower BMI values, lower fat mass percentage, lower FFM index, and lower FM index compared to low-risk patients. However, an interesting finding was that a great percentage of low-risk patients (74.5%) were sarcopenic obese, whereas the prevalence of sarcopenic obesity was 31.2% in medium/high-risk patients. This result highlights the urgent need for body composition analysis of these patients since other methods of nutritional assessment are not very sensitive to capturing alterations in lean and adipose tissue mass.

Body composition, especially low levels of lean mass, has been linked to vitamin D status [28]. In our study, 92.2% of patients were 25-OH-D deficient. Thalassemia is considered to be one of the chronic diseases associated with an increased risk of vitamin D deficiency. The prevalence of vitamin D deficiency is particularly seen in β-thalassemia major patients on repeated blood transfusion. A previous study reported that only 18% of a sample of 361 patients with thalassemia residing in North America had sufficient levels of 25-OH vitamin D [29]. Similar levels of inadequacy have been also observed in other studies [30,31]. Regarding the general population, estimates of the prevalence of 25(OH)D levels have been reported as 40% in Europe, with lower levels observed in childhood and the elderly, while European Caucasians tend to show lower rates of vitamin D deficiency compared with nonwhite individuals [12,32]. Up to 80% of the vitamin D requirement is synthesized in the skin, and only a small proportion is obtained from dietary sources. Interestingly, Goldberg et al. indicated that nearly 100% of subjects enrolled in their study consumed inadequate vitamin D, with over a third presenting with depressed circulating levels [20]. Defective synthesis of 25 OH vitamin D and/or hypoparathyroidism have been observed in these patients. Decreased outdoor activities in thalassemic patients can also compromise the cutaneous synthesis of vitamin D [33]. Timely high-dose vitamin D supplementation of thalassemic patients could replenish vitamin D levels and potentially improve the quality of life of these patients [34].

However, in this study, there are some limitations that should be addressed. First, the number of patients was relatively small, so the statistical significance of some associations could have been lost. Second, it was a cross-sectional, single-center study that was not able to determine cause-and-effect relationships between different variables. Moreover, in our study, we did not include a control group in order to compare our findings with healthy individuals, especially in terms of body composition analysis. Last but not least, due to the variability between patients, our data may not be fully generalizable to all individuals with thalassemia.

## Conclusions

To conclude, adult patients with β-thalassemia major in our group had altered body composition, presenting with increased adiposity and significantly low levels of lean body mass. The prevalence of sarcopenic obesity was also very high in this group of patients. In addition, vitamin D deficiency is very common in transfusion-dependent thalassemic patients.

Timely detection of patients at risk could lead to the prioritization of patients who could benefit from nutritional interventions. Multicenter studies should be conducted in order to define the best dietary approach for these patients in order to attenuate the negative effects of disease and treatments’ side effects.
